# HMGB1 Is a Potential Biomarker for Severe Viral Hemorrhagic Fevers

**DOI:** 10.1371/journal.pntd.0004804

**Published:** 2016-06-27

**Authors:** Katarina Resman Rus, Luka Fajs, Miša Korva, Tatjana Avšič-Županc

**Affiliations:** Institute of Microbiology and Immunology, Faculty of Medicine, University of Ljubljana, Ljubljana, Slovenia; University of Texas Medical Branch, UNITED STATES

## Abstract

Hemorrhagic fever with renal syndrome (HFRS) and Crimean-Congo hemorrhagic fever (CCHF) are common representatives of viral hemorrhagic fevers still often neglected in some parts of the world. Infection with Dobrava or Puumala virus (HFRS) and Crimean-Congo hemorrhagic fever virus (CCHFV) can result in a mild, nonspecific febrile illness or as a severe disease with hemorrhaging and high fatality rate. An important factor in optimizing survival rate in patients with VHF is instant recognition of the severe form of the disease for which significant biomarkers need to be elucidated. To determine the prognostic value of High Mobility Group Box 1 (HMGB1) as a biomarker for disease severity, we tested acute serum samples of patients with HFRS or CCHF. Our results showed that HMGB1 levels are increased in patients with CCHFV, DOBV or PUUV infection. Above that, concentration of HMGB1 is higher in patients with severe disease progression when compared to the mild clinical course of the disease. Our results indicate that HMGB1 could be a useful prognostic biomarker for disease severity in PUUV and CCHFV infection, where the difference between the mild and severe patients group was highly significant. Even in patients with severe DOBV infection concentrations of HMGB1 were 2.8–times higher than in the mild group, but the difference was not statistically significant. Our results indicated HMGB1 as a potential biomarker for severe hemorrhagic fevers.

## Author Summary

Dobrava virus (DOBV), Puumala virus (PUUV) and Crimean-Congo Hemorrhagic fever virus (CCHFV) are important causative agents of viral hemorrhagic fevers. Clinical severity of Hemorrhagic fever with renal syndrome (HFRS) and Crimean-Congo Hemorrhagic fever (CCHF) varies greatly from a mild, nonspecific febrile illness to a severe form of the disease with hemorrhaging and high fatality rate. In this study we investigated the role of High Mobility Group Box 1 (HMGB1) as a prognostic marker for the severe form of HFRS and CCHF. The study showed high concentration of HMGB1 in HFRS and CCHF patients in comparison with healthy donors. Even more, higher levels of HMGB1 were measured in patients with the severe form of diseases. Interestingly, patients with severe PUUV infection had the highest concentration of HMGB1, although infection with DOBV and CCHFV usually causes more severe hemorrhagic manifestations. It is clear that HMGB1 has a broad repertoire of immunological functions and is involved in various pathways of immunity, inflammation and cancer progression, therefore also the induction by infection with PUUV, DOBV or CCHFV can influence different pathways. Nonetheless our research demonstrates a potential use of HMGB1 as an important biomarker for severity in PUUV and CCHFV infection.

## Introduction

Viral hemorrhagic fevers (VHFs) are characterized by a severe multisystem syndrome and are caused by viruses from several different families. Among them, hantaviruses and Crimean-Congo Hemorrhagic fever virus (CCHFV), members of the *Bunyaviridae* family, represent important causative agents of VHFs. Hantaviruses are present globally and each hantavirus is closely related to a specific rodent or insectivore natural host. Humans are generally infected by inhaling virus-contaminated aerosols from small mammal’s excreta. Hantaviruses cause two typical syndromes: Hemorrhagic fever with renal syndrome (HFRS) in Europe and Asia, and Hantavirus cardiopulmonary syndrome in the Americas. HFRS is an endemic disease in Slovenia, caused by Dobrava virus (DOBV) and Puumala virus (PUUV). PUUV usually causes a milder form of the disease, called nephropathia epidemica (NE), whereas DOBV is mainly responsible for the more severe disease with up to 10% case fatality rate. However, clinical severity of HFRS varies greatly. In Slovenia, both severe and mild clinical courses of the disease have been observed, with an overall case fatality rate of 4.5% [1,2]. CCHFV is the causative agent of Crimean-Congo hemorrhagic fever (CCHF), which is the most widespread tick-borne viral infection. CCHF virus is transmitted by several genera of ixodid ticks to a variety of wild and domestic mammals, which develop a transient viremia without any signs of the illness. Humans are infected with CCHFV through a tick bite or exposure to the blood or other bodily fluids of infected animals or patients [3]. The course of the disease can be extremely severe, with hemorrhaging and case fatality rate between 3% and 30%. Similarly to HFRS, CCHF can also result in a mild, nonspecific febrile illness or an asymptomatic infection [4].

Studies showed that DOBV, PUUV and CCHFV infections cause different intensity of clinical and pathological manifestations in patients, like increased vascular permeability, coagulopathy, thrombocytopenia and hemorrhages. Immuno-pathogenic factors play an important role in the pathogenesis of both HFRS and CCHF. It was shown that the fatal cases of CCHF had an elevated serum cytokine level, weak or absent antibody response and high viral load [5,6]. Interferon (IFN) response also plays an important role in the pathogenesis of both diseases. Early activation of the type I IFN response was delayed by pathogenic hantaviruses and CCHFV, thereby enabling early viral replication and the spread of infection [7,8,9].

During the acute phase of HFRS and CCHF, patients show typical laboratory parameters like anemia, leukocytosis, thrombocytopenia, proteinuria, hematuria, elevated liver enzymes and serum creatinine. Severity of the disease is also linked to the levels of different biomarkers and can serve as prognostic indicators. Biological markers like interleukin 6, pentraxin-3, idoleamine 2,3 dioxygenase, soluble urokinase-type plasminogen activator receptor and GATA-3 were found to correlate with severity of NE [10,11,12]. C-reactive protein (CRP) and procalcitonin (PCT) are used particularly for discrimination between bacterial and viral infections and are usually found in high levels in patients with systemic bacterial infections and were also highly elevated in HFRS and CCHF patients [13,14,15,16].

Another proinflammatory marker connected to prognosis of hemorrhagic fevers is High Mobility Group Box 1 (HMGB1). HMGB1 is a non-histone nucleosomal protein that binds and bends DNA. It functions as a nuclear remodeling factor [17,18]. HMGB1 is usually found in the cell nucleus and can be secreted into the extracellular milieu passively from necrotic cells or actively by activated immune cells [19,20]. HMGB1 was linked to play a potential role in sepsis [21], hemorrhagic shock, trauma [22] and several infectious viral diseases [23,24,25]. Allonso and coworkers showed that HMGB1 can be used as a biomarker for severe dengue prognosis and high HMGB1 levels were correlated with increased vascular permeability, intensity of symptoms and incidence of secondary infection [26,27].

Although different clinical studies were conducted, complete immunopathogenesis of aforementioned VHFs is still unknown. The aim of our study was to investigate the role of HMGB1 as a prognostic marker for HFRS and CCHF.

## Methods

### Ethics statement

The study was approved by Republic of Slovenia National Medical Ethics Committee (69/03/12 and 30/04/15). Collecting of CCHF serum samples was part of the CCH Fever network (Collaborative Project) supported by the European Commission under the Health Cooperation Work Programme of the 7^th^ Framework Programme (grant agreement no. 260427). All participants gave an oral and written informed consent. The study was conducted according to the principles expressed in the Declaration of Helsinki.

### Patients

Altogether, 128 serum samples of patients with hemorrhagic fever were included in this study. HFRS patients were hospitalized in different Slovenian hospitals and clinical diagnosis was confirmed with serological or molecular tests as described elsewhere [1]. From 72 HFRS patients included in the study, 24 were infected with DOBV and 48 were infected with PUUV. Additionally, we have analyzed 29 serial samples from 7 HFRS patients. Fifty-six patients from Kosovo with confirmed CCHF were also included in the study. CCHFV infection was confirmed as described before [6]. For each patient a detailed medical chart was collected and significant laboratory parameters were analyzed. Additionally, serum samples of 61 healthy donors were tested. Blood samples from healthy donors were processed as patients’ samples.

### HMGB1 assay

Concentration of HMGB1 was measured with commercially available HMGB1 capture enzyme-linked immunosorbent assay (ELISA) kit (Chondrex, USA) according to the manufacturer’s instructions. Samples were measured in duplicate.

### Statistical analyses

Statistical analyses were performed using GraphPad Prism version 6 for Windows (GraphPad Software, San Diego, CA, USA). To analyze the normal distribution of data the D’Agostino-Pearson normality test was performed. Differences between groups were calculated using the non-parametric Mann-Whitney U test and Kruskal Wallis analyses. All statistical tests were two-tailed. A *p*-values below 0.05 was considered statistically significant.

## Results

### Patients and clinical data

To determine HMGB1 dynamic in the acute stage of illness multiple sample per patients were tested. Twenty-nine samples from 7 patients with HFRS were analyzed. Our results showed that in all 7 patients, the concentration of HMGB1 was the highest in the first sample and that the concentration of HMGB1 decreased during the hospitalization (supplement data, S1 Fig). According to the observed kinetic, we have further evaluated the HMGB1 concentration in the first available serum sample of patients with hemorrhagic fever.

Seventy-two patients with confirmed HFRS (24 infected with DOBV and 48 with PUUV) and 56 patients with confirmed CCHF were enrolled in the study. Patients’ serum samples were collected during the onset of disease, at the admission to the hospital. Samples of HFRS patients were collected in the mean on 2.5 day of hospitalization (between 2^nd^ and 18^th^ day of illness). Specifically, samples of DOBV infected patients were collected in the mean on 2.8 day of hospitalization (between 3^rd^ and 18^th^ day of illness) and samples of PUUV infected patients were collected in the mean on 2.4 day of hospitalization (between 2^nd^ and 11^th^ day of illness). Similar as in HFRS patients, samples of CCHF patients were collected in the mean on 2.9 day of hospitalization (between 1^st^ and 21^st^ day of illness). Patients were categorized into two groups: mild and severe, based on disease severity. Fatal patients were included in the severe group. Patients with HFRS were categorized based on clinical data and laboratory parameters as described before [28,29]. Twelve patients infected with DOBV were categorized as having the severe form of the disease and 12 as the mild form. Twenty-three PUUV patients were categorized as severe and 25 as mild. Patients with CCHF were categorized based on the classification by Swanepoel et al. [6,30]. Twenty-eight were categorized as having the severe form of the disease (13 fatal) and 28 as the mild.

### HMGB1 in hemorrhagic fevers

To examine whether the demographic influenced concentration of HMGB1, we have analyzed the age and gender composition of patients with hemorrhagic fevers. Among all included HFRS patients 81% were male and 19% were female. Age distribution among patients with HFRS was between 20 and 49 years. Thirty-nine male patients (70%) and 17 female patients (30%) with CCHF were included in our study. Most CCHF patients were aged between 30 and 39 years (18%). Age and gender composition of the control group were similar than the study group. Our results indicated no significant difference in concentration of HMGB1 among different age groups (for HFRS patients p = 0.95 and for CCHF patients p = 0.82) or genders (for HFRS patients p = 0.84 and for CCHF patients p = 0.34) in patients with hemorrhagic fevers (supplement data, S2 and S3 Figs).

**Fig 1:**
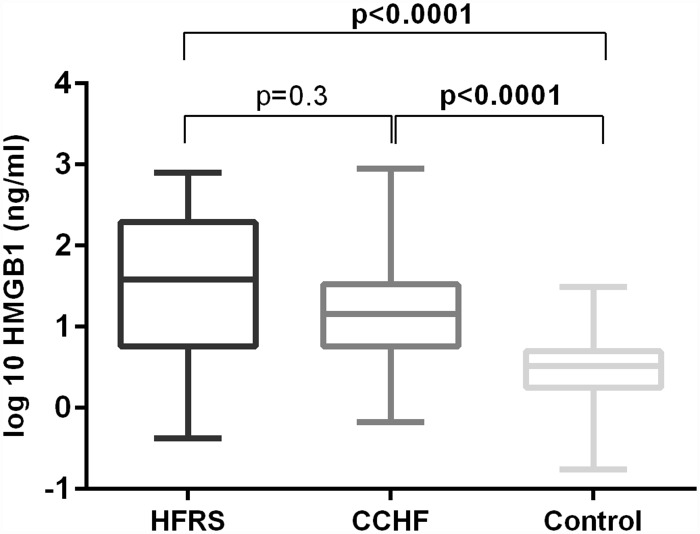
Comparison of serum HMGB1 concentrations in patients with HFRS, CCHF and healthy controls. The Kruskal Wallis test was used for statistically analyses. A *p*-values below 0.05 were considered statistically significant.

To investigate whether HMGB1 concentration is elevated in patients with hemorrhagic fever, we compared HMGB1 levels between 72 patients with HFRS, 56 patients with CCHF and 61 healthy donors. Results show that HMGB1 concentrations were statistically significantly higher in patients with HFRS and CCHF than in healthy donors (p<0.0001, Fig 1). The median concentrations were 11.6–times and 4.4–times higher in patients with HFRS and CCHF than in healthy donors (Table 1). The median concentration of HMGB1 was 2.6–times higher in patients with HFRS than in patients with CCHF, but the difference was not statistically significant (p = 0.3). These results indicated that HMGB1 concentration is elevated in patients with hemorrhagic fevers.

**Table 1:** HMGB1 concentration in patients with hemorrhagic fevers.

	HFRS patients	DOBV severe and fatal	DOBV mild	PUUV severe	PUUV mild	CCHF patients	CCHF severe and fatal	CCHF mild	Control
Number of sera	72	12	12	23	25	56	28	28	61
Median HMGB1 concentration (ng/ml)	38	45,7	16	138	7,3	14,5	19	11,3	3,3
Minimal to maximal HMGB1 concentration (ng/ml)	0,4–787	0,4–787	1–493	4,8–702	0,8–513	0,7–897	0,7–897	1,4–75	0,2–31

### HMGB1 level in patients with CCHF

**Fig 2:**
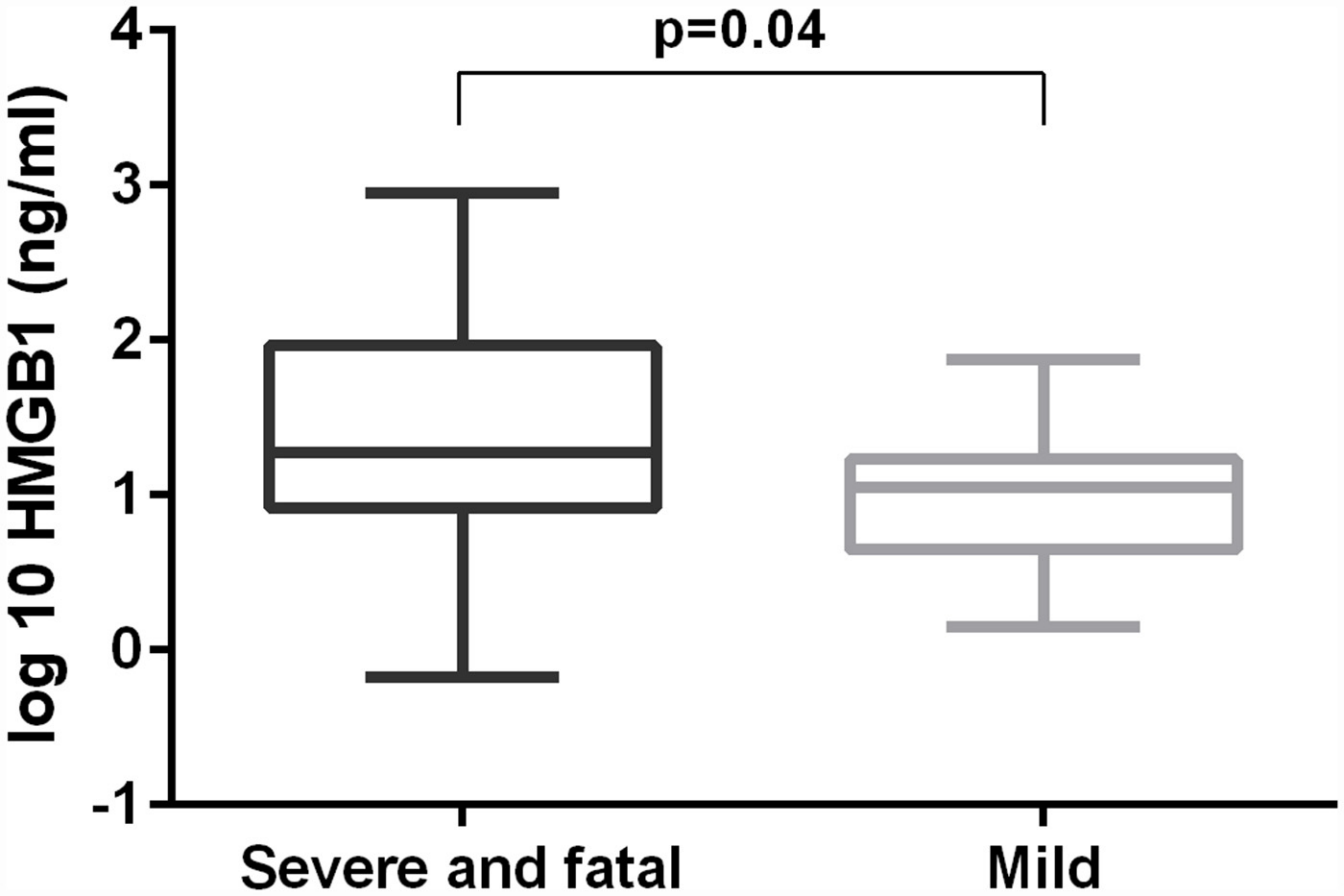
Comparison of serum HMGB1 concentrations in patients with CCHFV infection according to the clinical course of the disease. The Mann-Whitney U test was used for statistically analyses. A *p*-values below 0.05 were considered statistically significant.

To determine whether HMGB1 concentrations are higher in the severe form of the disease, we compared the levels in both groups. Concentration of HMGB1 was significantly higher in patients with the severe form of CCHF than in patients with the mild form of the disease (p = 0.04, Fig 2), although the median HMGB1 concentration was only 1.7–times higher in patients with the severe form of the illness than in patients with the mild form of the disease (Table 1). These results show that patients with severe CCHF had significantly elevated levels of HMGB1 compared to patients with the mild form of the disease.

### HMGB1 level in patients with HFRS

**Fig 3:**
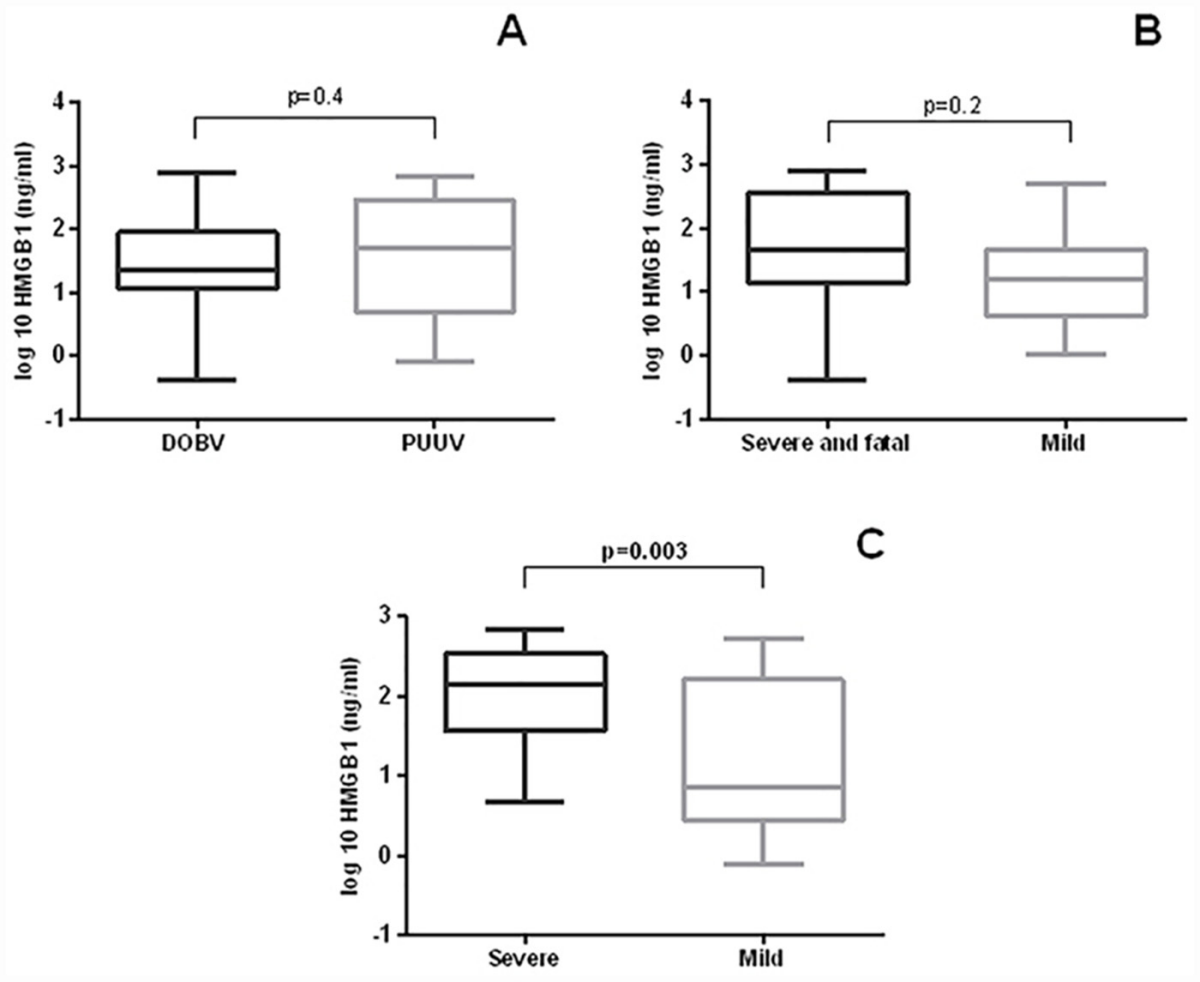
**A** Comparison of serum HMGB1 concentrations in patients with DOBV and PUUV infection. The Mann-Whitney U test was used for statistically analyses. A *p*-values below 0.05 were considered statistically significant. **B** Comparison of serum HMGB1 concentrations in patients with DOBV infection according to the clinical course of the disease. The Mann-Whitney U test was used for statistically analyses. A *p*-values below 0.05 were considered statistically significant. **C** Comparison of serum HMGB1 concentrations in patients with PUUV infection according to the clinical course of the disease. The Mann-Whitney U test was used for statistically analyses. A *p*-values below 0.05 were considered statistically significant.

To determine if there is a difference in concentration of HMGB1 between HFRS patients, we compared levels of HMGB1 in patients infected with DOBV and PUUV. The median HMGB1 concentration was 2.2–times higher in patients infected with PUUV than in patients infected with DOBV, but the difference was not statistically significant (p = 0.4, Fig 3a).

Next, we assessed whether the HMGB1 concentrations were higher in patients with severe HFRS. The median HMGB1 concentration was 2.8–times higher in patients with severe DOBV infection than in patients with mild DOBV infection (Table 1), but the difference was not statistically significant (p = 0.2, Fig 3b). Comparison of patients with severe and mild PUUV infection showed high differences between groups. The median HMGB1 concentration was 19–times higher in patients with severe PUUV infection than in patients with mild PUUV infection and the difference was statistically significant (p = 0.003, Fig 3c). These results clearly showed that patients with severe PUUV infection had the highest HMGB1 concentration (Table 1).

## Discussion

Multiple studies have aimed to identify prognostic biomarkers for hemorrhagic fever in order to prevent a fatal outcome of the disease. It was found that CRP and PCT are elevated in patients with HFRS and CCHF, but these biomarkers are not specific and not sufficient to predict virus infection severity. However, recently it was shown that HMGB1 can be used as a biomarker for Dengue virus (DENV) diagnosis and disease prognosis [27]. In this study we focused our investigation on HMGB1 as a possible prediction biomarker for disease severity of HFRS and CCHF.

Our investigation included patients’ samples from the acute phase of the disease, as previous reports and our results showed that HMGB1 concentrations are higher during the first days of the illness [26]. Our results indicated that age and gender distribution have no influence on concentration of HMGB1 in patients with hemorrhagic fevers. Obtained results indicated that concentrations of HMGB1 are higher in patients with HFRS and CCHF than healthy controls, however, they were similar between the two VHFs. Elevated levels of HMGB1 were anticipated in patients with hemorrhagic fevers, since it was shown that HMGB1 has a role in many diseases, including infectious viral diseases [23,24,25]. It was shown that released HMGB1 can function as a proinflammatory cytokine. According to that, our results indicated that HMGB1 is likely to be involved in the immunopathogenesis of hemorrhagic fevers.

To determine the possible role of HMGB1 in the pathogenesis of studied VHFs and to ascertain its probable prognostic value, we compared concentrations of HMGB1 between patients with severe and mild CCHF. Our results demonstrated statistically higher levels of HMGB1 in patients with the severe clinical course. This implies that the level of HMGB1 might play an important role in the immunopathogenesis of CCHF and could probably be used for disease severity prediction.

Furthermore, we compared patients with DOBV and PUUV infection, as it is generally believed that DOBV is mainly responsible for more severe HFRS cases. Patients infected with PUUV indicated higher levels (although not statistically significant) of HMGB1 than patients infected with DOBV. To determine, if the level of HMGB1 can serve as a prognostic marker in HFRS, we compared concentrations of HMGB1 between patients with severe and mild clinical courses. Patients with the severe DOBV infection had higher HMGB1 levels than patients with the mild course of the disease. Results were not statistically significant, which indicated that HMGB1 is not an appropriate biomarker for disease severity prediction in DOBV infections. On the contrary, our results demonstrated that HMGB1 can serve as a suitable biomarker for prognosis of severe PUU infections, considering that patients with severe PUUV infection had significantly higher levels of HMGB1 than patients with the mild disease form.

Our results suggested that higher HMGB1 levels are a general feature of severe forms of viral hemorrhagic fevers. Patients with the severe PUUV infection indicated the highest HMGB1 level among HFRS and CCHF patients, although infection with DOBV and CCHFV usually cause more severe hemorrhagic manifestations than PUUV. One of the possible explanations for high levels of HMGB1 in severe PUUV infected patients could be the inhibition of an early innate immune response. Immunological studies revealed that pathogenic hantaviruses and CCHFV suppress the early IFN-β response. Ability of hantaviruses to inhibit innate immunity vary in their degrees of pathogenicity. It was shown that both DOBV and PUUV inhibit IFN-β induction, but the inhibition reported for PUUV was only 10–30% of IFN responses reduction [31]. On the other hand it was demonstrated that IFN-β and STAT-1 have critical and essential roles in HMGB1 release [32]. Additionally, it was shown that release of HMGB1 is dependent on the activation of the JAK/STAT1 pathway by IFN-β [33]. This could probably explain why HMGB1 levels are higher in patients with the severe PUUV infection, although DOBV causes a more severe HFRS than PUUV. Inhibition of IFN-β by DOBV virus and consequently lower levels of HMGB1 could be the reason why differences between severe and mild DOBV infections were not statistically significant. On the contrary, patients with severe CCHF indicated statistically significantly higher level of HMGB1 than patients with the mild infection, although it was shown that CCHFV suppresses IFN-β promoter mediated gene expression [8]. HMGB1 also plays an important role in hemorrhagic shock as it can be released passively into the extracellular milieu from damaging cells and functions as an inflammatory cytokine [34]. This indicates that abundant bleeding could be one of the possible reasons for elevated HMGB1 levels in patients with severe CCHF. Both, DOBV and CCHFV can cause severe forms of the disease with bleeding manifestations, however the initial manifestation in patients with severe CCHF more commonly progresses to large cutaneous ecchymoses and bleeding from the gastrointestinal and urinary tracts [30]. Nonetheless, HMGB1 has a broad repertoire of immunological functions and is involved in different pathways of immunity, inflammation and cancer progression. Additional *in vivo* and *in vitro* studies must be conducted to confirm all roles of HMGB1 in pathogenesis of VHFs.

In conclusion, our study is the first to represent that HMGB1 levels are increased during hemorrhagic fevers caused by CCHFV, DOBV and PUUV. The work presented here indicates elevated HMGB1 levels in patients with severe HFRS and CCHF. Our research demonstrated potential use of HMGB1 as a biomarker for severity in PUUV and CCHFV infections. The induction and release of HMGB1 is very complex and is involved in different pathways of the host immune system. Because of immune-mediated response of HMGB1 further immunological studies are needed to clarify, whether HMGB1 can be used as a prognostic marker for hemorrhagic fever outcomes.

## Supporting Information

S1 Fig Kinetics of HMGB1 in serial samples from HFRS patients. Each graph represents one patient.
(TIF)

S2 Fig HMGB1 concentration in HFRS and CCHF patients according to the gender distribution. (TIF)

S3 Fig HMGB1 concentration in HFRS and CCHF patients according to the age distribution. (TIF)
