# Supplementary figures and images for: HMGB1 Is a Potential Biomarker for Severe Viral Hemorrhagic Fevers
Source: PLoS Negl Trop Dis. 2016 Jun 27;10(6):e0004804. doi: 10.1371/journal.pntd.0004804 (PMC4922654; doi:10.1371/journal.pntd.0004804)

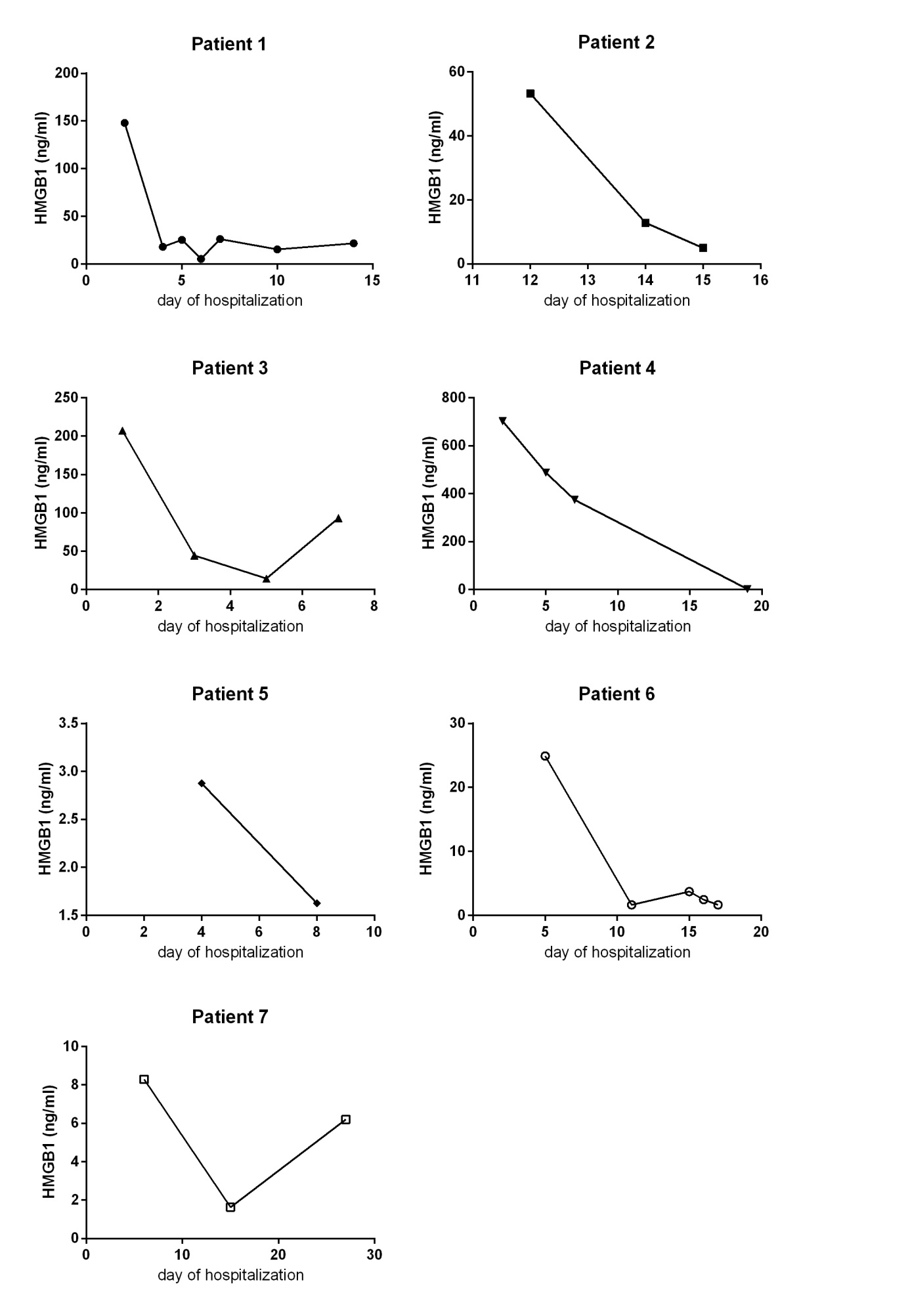

Supplement: S1 Fig — Each graph represents one patient. (TIF) [file pntd.0004804.s001.tif]

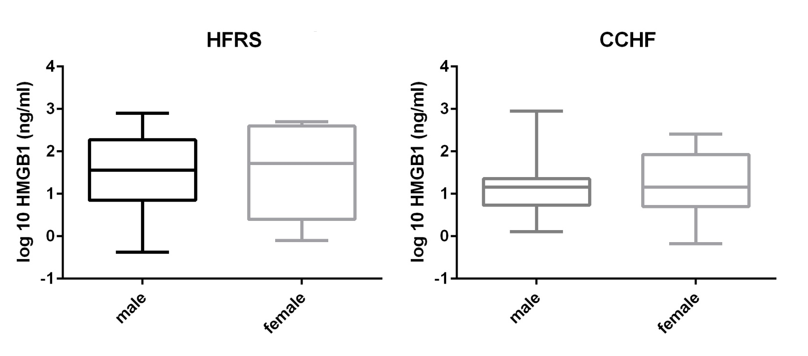

Supplement: S2 Fig — (TIF) [file pntd.0004804.s002.tif]

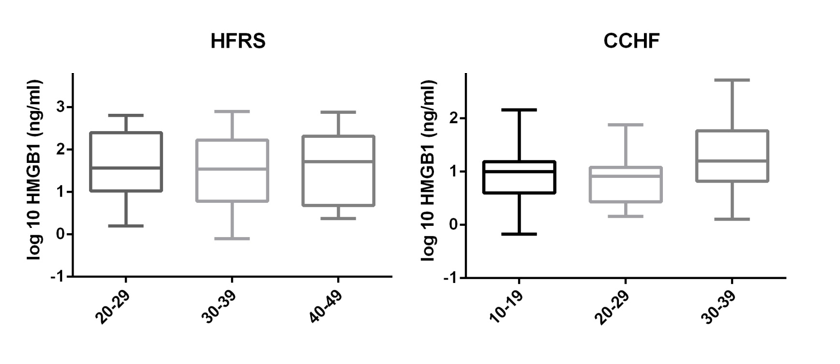

Supplement: S3 Fig — (TIF) [file pntd.0004804.s003.tif]
